# Measurements of Methane Emissions from a Biofertilizer Storage Tank Using Ground-Based Hyperspectral Imaging and Flux Chambers

**DOI:** 10.1021/acs.est.3c06810

**Published:** 2024-02-14

**Authors:** Magnus Gålfalk, Sören Nilsson Påledal, Johan Yngvesson, David Bastviken

**Affiliations:** † Department of Thematic Studies − Environmental Change, Linköping University, Linköping 581 83, Sweden; ‡ Tekniska Verken AB, Linköping 581 15, Sweden; § RISE Research Institutes of Sweden, Goteborg 412 58, Sweden

**Keywords:** methane, greenhouse gas, emissions, hyperspectral imaging, flux chambers, biofertilizer storage, visualization, unknown sources

## Abstract

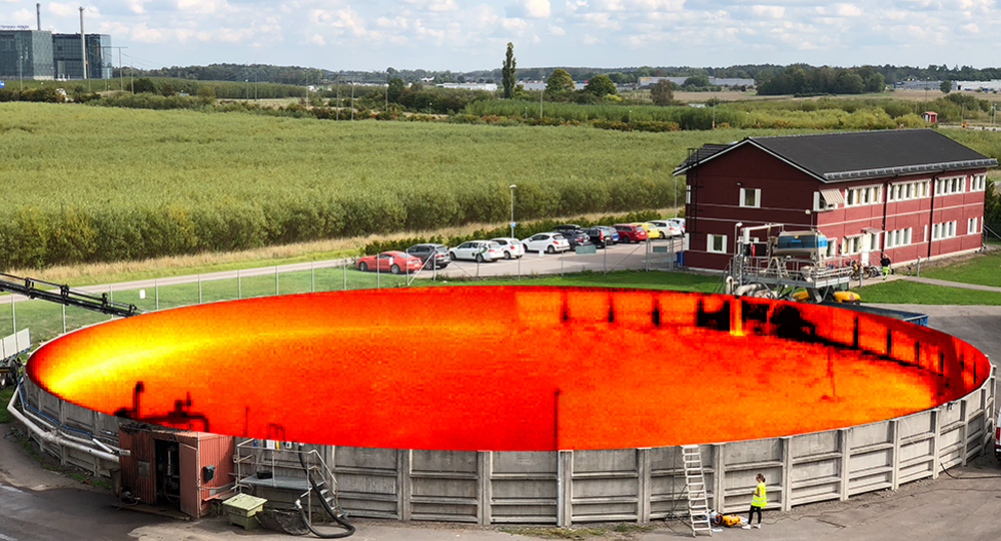

Open storages of organic material represent potentially large sources of the greenhouse gas methane (CH_4_), an emissions source that will likely become more common as a part of societal efforts toward sustainability. Hence, monitoring and minimizing CH_4_ emissions from such facilities are key, but effective assessment of emissions without disturbing the flux is challenging. We demonstrate the capacity of using a novel high-resolution hyperspectral camera to perform sensitive CH_4_ flux assessments at such facilities, using as a test case a biofertilizer storage tank for residual material from a biogas plant. The camera and simultaneous conventional flux chamber measurements showed emissions of 6.0 ± 1.3 and 13 ± 5.7 kg of CH_4_ h^–1^, respectively. The camera measurements covered the whole tank surface of 1104 m^2^, and the chamber results were extrapolated from measurements over 5 m^2^. This corresponds to 0.7–1.4% of the total CH_4_ production at the biogas plant (1330 N m^3^ h^–1^ corresponding to 950 kg h^–1^). The camera could assess the entire tank emission in minutes without disturbing normal operations at the plant and revealed additional unknown emissions from the inlet to the tank (17 g of CH_4_ h^–1^) and during the loading of the biofertilizer into trucks (3.1 kg of CH_4_ h^–1^ during loading events). This study illustrates the importance of adequate measurement capacity to map methane fluxes and to verify that methane emission mitigation efforts are effective. Given the high methane emissions observed, it is important to reduce methane emissions from open storage of organic material, for example by improved digestion in the biogas reactor, precooling of sludge before storage, or building gastight storage tanks with sealed covers. We conclude that hyperspectral, ground-based remote sensing is a promising approach for greenhouse gas monitoring and mitigation.

## Abstract 2

Emissions from biogas production contributes to global warming. Our method allowed quick full-scale emission mapping and visualization of methane with previously unknown sources observed.

## Background

To reach societal climate goals, greenhouse gas (GHG) emissions need to be identified and measured to enable mitigation and verification that efforts to reduce emissions are effective. Open storage of organic material, such as sludge, manure, and other types of biofertilizers can be important sources of GHG emissions.^1−4^ Their abundances are likely to increase because societies need to increase the nutrient and energy recycling from organic material in the efforts toward sustainability. However, measuring GHG emissions from such storage is presently a challenge. The stored material can have variable characteristics in terms of the organic matter composition and viscosity. The GHG emissions are affected by the microbial formation rates of carbon dioxide (CO_2_), methane (CH_4_), and nitrous oxide (N_2_O), in combination with gas transport processes influenced by turbulence in fluid and cracks in dry material types leading to increased air exposure and consequent gas exchange. Exposure to wind, deliberate mixing, or aeration, as well as surface disturbances from measurement equipment, has the potential to influence gas fluxes. Therefore, traditional methods using various types of enclosures that may change the wind exposure or cause physical disturbance of the surfaces should be supplemented by noninvasive approaches to measure gas fluxes. Here, we present such a comparison between traditional enclosure-based and new noninvasive hyperspectral gas imaging techniques to measure CH_4_ emissions from a biofertilizer tank, where digestate from biogas production is stored awaiting use as farmland fertilizer. Such open biofertilizer storage has been identified as a main source of methane emissions, and emissions of several percent of the total associated biogas production have been measured. A study of 69 biogas plants in Denmark^5^ found CH_4_ losses in the range 0.3–40.6%, and plants using open digestate storage, instead of covered gastight storage with gas collection and utilization, were found to be major CH_4_ emitters. In a recent study of the biogas supply chain,^6^ it was found that CH_4_ emissions were underestimated, with digestate storage being the largest emission source.

Currently, CH_4_ fluxes from open areas of biofertilizer storage are frequently measured by using flux chambers, giving accurate flux estimates of m^2^-sized footprints. Flux chamber estimates of methane emissions require a large number of measurements to be representative of the entire storage area, as spatial variability is often high. Thus, a primary limitation of chamber-based measurement approaches is the significant investment in effort and time required to obtain reliable emissions estimates. It can also be unclear how much of the variability is real spatial variability versus experimental measurement uncertainty (or variability) associated with the physical contact between a chamber and the biofertilizer surface.

Alternative methods include the tracer dispersion method, which uses a controlled tracer gas release at the source area and assumes that this gas will disperse in the same way as the emitted CH_4_. Concentration measurements of both CH_4_ and the tracer gas can then be made downwind of a biogas plant to estimate the total CH_4_ emission (see ref (7) for a method description). The inverse dispersion modeling method, a micrometeorological method that estimates the CH_4_ emission using wind and downwind CH_4_ concentration measurements, simulating the motion of air packages back in time for source-attribution,^8^ can also be used. Both methods have uncertainties regarding source footprints, and it can be challenging to separate different local sources within a biogas plant. Drone-based mass balance calculations (fixed wing and rotary) represent a new method for calculating total emissions on a small to large scale that has made fast progress in recent years due to the development of low-weight sensors (see ref (9) for a review) and could potentially be used in the future at biogas plants. There is also a common optical method for finding potential sources using narrow-band infrared cameras (such as the FLIR GF320) with high sensitivity in the spectral range 3.2–3.4 μm, which contains strong CH_4_ absorption features. It is a quick approach for finding leaks large enough to make the CH_4_ signal dominate signals from other gases absorbing light in this spectral region. However, at low to medium CH_4_ levels, interferences from other gases including H_2_O are challenging to distinguish and quantification of emissions is thereby challenging with the narrow-band infrared camera approach.

Another optical method for ground-based remote sensing is so-called hyperspectral imaging, which can be used to simultaneously visualize and calculate emissions of specific gases. It has the advantage of having high spectral resolution, thereby distinguishing separate gases and potentially discovering unknown fluxes anywhere in the field of view. Because, e.g., H_2_O and other target gases can be separated, the target gas concentrations can be combined with water vapor motion representing air movement in the same set of camera data, in turn allowing flux estimates in post processing without the need of accessory data. We previously described a hyperspectral method, specializing in high sensitivity for CH_4_ using a customized Telops camera (Hypercam methane), our in-house written software, and the strongly absorbent 7.7 μm band (Gålfalk et al.).^10^ Since then, we have added a LiDAR distance scanner making background distance maps of the instrument’s field of view, further increasing the CH_4_ assessment accuracy, and used this approach to assess emissions from different treatment steps at wastewater treatment plants (Gålfalk et al).^23^

In the current study we (a) extend the general potential use of this technique for remote assessment of ring-tank CH_4_ emissions and (b) provide novel measurements of CH_4_ emissions from biofertilizer storage, comparing the hyperspectral camera method and flux chamber measurements. The pros and cons of both methods were investigated, and the possibility to discover unknown sources at the site was also tested.

## Methods

The biofertilizer storage tank, serving as a case for this study, had an inner diameter of 37.5 m (surface area of 1104 m^2^) and a depth of 4 m, with a maximum storage volume of 4000 m^3^. During our measurements, the storage tank was filled to 2/3 of its maximum capacity, corresponding to about 2500 m^3^ of biofertilizer material, and the biogas plant produced 950 kg CH_4_ h^–1^. In order to evaluate the CH_4_ emissions from the biofertilizer storage tank relative to the total production of the biogas plant, production data were used for a time period of 14 days before our measurements (August 22 through September 4, 2018), retrieved from the control system of the facility, where data are stored continuously.

### Remote Sensing

Our hyperspectral method is based on spectroscopic data from an imaging Fourier transform spectrometer (iFTS). It can be described as a camera that in addition to a 2D image generates a light spectrum for each pixel, and in each spectrum the absorbance of some gases present in the air between the background and the camera can be used to quantify gas concentrations. In the present case, an infrared (IR) spectrum around the CH_4_ absorption at 7.7 μm was used. The aim of our use of the iFTS was to detect, visualize, and measure gas fluxes in any scene. Method details are provided elsewhere (e.g., Gålfalk et al.^10,11^). Briefly, a requirement of this passive infrared method is a temperature difference between the background and the gas being measured, with larger differences giving better sensitivities. Data processing then goes from raw data (thousands of IR exposures per minute) to aggregated spectra (one for each coordinate in an image) and finally to maps of the background temperature and average gas concentrations for different gases across the whole scene covered by the image exposures. Additional maps across the imaged scene used in the flux calculations are a background distance map (using a scanning LiDAR on top of the instrument) and air speed perpendicular to the camera (calculated from how quickly water vapor features move between sets of images). In the final step, a gas flux is calculated from different parts of the imaged scene by comparing in- and outgoing gas amounts around each part of the scene. This mass balance step thus combines a gas content map and air motion.

Measurements were made for 4.5 h (10:42–15:12) on the fifth of September 2018. The hyperspectral camera produced a large amount of data, with 320 × 256 spectra every 30th second. The data (also called data cubes) can be used in two ways: (1) to calculate wind velocities from video sequences of the frames (corrected for the interference which Fourier transform spectrometry is based on) and (2) calculation of column density maps (ppm·m) of CH_4_ and H_2_O, as well as background and air temperatures, from modeling of the obtained spectra for each pixel as described in detail elsewhere.^11,12^ Mounted on top of the hyperspectral camera was a customized LiDAR (DST Control AB), simultaneously mapping background distances in the same field of view as the hyperspectral camera (Figure S1).

The hyperspectral method is passive, meaning that it is based on a temperature difference between the background and gas temperature. Background temperatures could be the biofertilizer surface in the storage, its edge, or the adjacent environment. A temperature difference of at least 1 °C is needed for reliable measurements, which is often the case except during rain, fog, or completely overcast weather. During our measurements, the weather was partly cloudy, giving a temperature contrast in the range of 2–12 °C between the air temperature and most of the background (including sludge material and tank walls), avoiding using the small areas of the background that had lower thermal contrast. The instrument field of view is 25 × 20 deg, which in this case meant that two scenes were needed to map the extent of the entire storage tank. Measurements were made by alternating between the two areas (white rectangles 3 and 4 in Figure S1), performing 16 measurements of 30 s for each area at a time.

The hyperspectral camera yielded high-resolution spectra that allowed modeling of gas column densities (Figure S3), making it possible to map the extended CH_4_ emissions and to quantify the corresponding fluxes. From mass balance calculations, combining column density maps and wind speed obtained from the camera data and a distance map from the LiDAR, CH_4_ fluxes were calculated. For a detailed description of the method, from spectroscopic modeling to flux calculation, see Gålfalk et al.^11^ We also used a weather station (Vaisala) for wind speed and direction comparisons, placed on the ground in an open space outside the tank. While the weather station provided a point reference measurement, wind speed measurements from air motion inside the tank using the camera videos are more suitable for the flux calculations as they capture local wind conditions in different parts of the tank. For the wind speed determination, we calculated the velocity of water vapor features across lines of pixels surrounding the tank (away from all the edges generating turbulence) over the measurement time periods, assuming that the average wind speed generated is a good representation of the air motion in our mass balance calculations for the same period.

For the mass balance calculations of gas emissions, only the time periods with steady winds across most of the tank and sufficient wind speed were used. Thereby, vertical air motion could be neglected over distances as short as the tank diameter. Air motion perpendicular to the camera line of sight, across the surface of the tank, were calculated from the same hyperspectral data that were used to calculate the spectra, but with interference patterns removed (i.e., following the movements of the strong combined signal of H_2_O and CH_4_ over time in the scene). To increase the signal-to-noise ratio in these calculations, we have averaged data of 30 consecutive exposures on a pixel-by-pixel basis. Then the air speed could be calculated from the motion of water vapor features between sets of pixels (i.e., two different times) and knowing the distance to the gas from the camera. Low-speed air motion toward or away from the instrument was neglected as the air will not move outside of the near or far edge of the volume used for mass balance, i.e., the volume from the camera lens to the background of the field of view.

**Figure 1:**
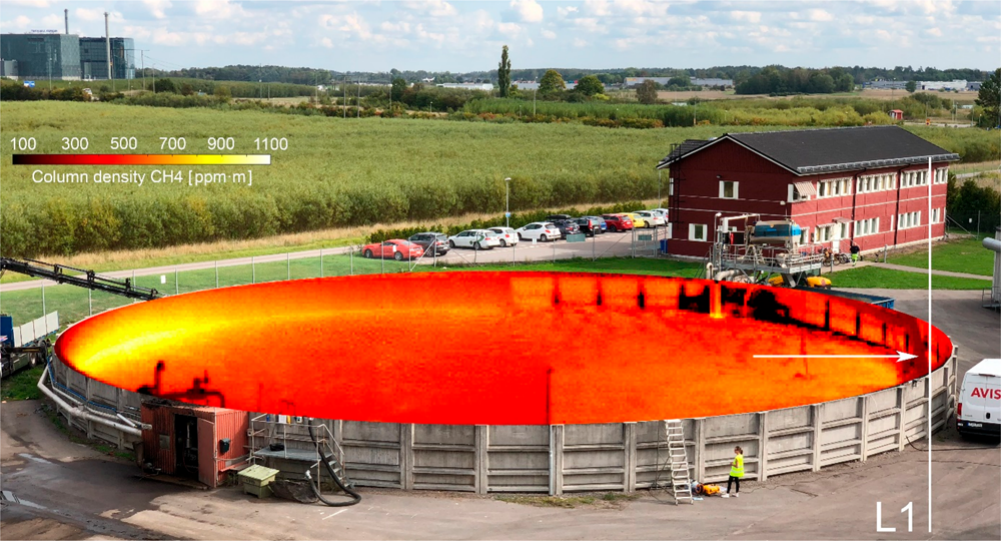
Biofertilizer tank with an overplotted CH_4_ column density map from the hyperspectral camera technique at a selected point in time for lines of sight with the tank as background. For visual clarity, we only show CH_4_ inside the tank, as this is where most of the gas is located, and the signal-to-noise ratios are the highest. Cross section L1 was used for quantification of total CH_4_ emissions during steady northern winds (toward the right) as emissions from the whole tank pass through this vertical area.

The CH_4_ mass balance calculations can be conducted in two ways using different approaches, depending on the atmospheric conditions and local meteorology. For stable winds with medium and high speeds (in relation to the tank diameter and rising speed of the air), the total tank emission can be calculated from the horizontal gas flux through a vertical area downwind of the tank (line L1 in Figure 1) as the emitted CH_4_ from the whole tank will pass this vertical area. We made whole-tank flux calculations using 19 data cubes (14 min period) having stable winds to the right in Figure 1 across line L1, measuring all of the gas passing through the vertical plane outlined by line L1. For cases with very low wind speeds (in relation to the size of the emitting area being measured), the vertical motion of the rising gas may cause an uncertainty in the total flux due to gas potentially flowing above the top of line L1 if it is not high enough. Another uncertainty is possible variations in air speed close to the walls of the tank. In order to estimate such uncertainties, we also calculated the tank flux from an open area in the center of the tank for time periods having stable winds. For this calculation we used five data cubes (2.5 min; 5 × 30 s) having suitable wind conditions during and before the measurements. For both flux calculations, we used data with stable wind speed and direction perpendicular to the camera line of sight, avoiding times when trucks were loading biofertilizer material on the left side of the tank which could otherwise disturb the measurements due to potentially very high CH_4_ point emissions (see Results). The total emission uncertainty was estimated from variation in calculated fluxes due to the uncertainty in wind speeds obtained from the camera data (which is the dominating error source for the total flux). The range of wind speeds were found from estimates at several positions in the tank over time, involving the data cubes used in the mass balance calculation.

### Flux Chambers

Simultaneously with hyperspectral imaging, measurements were made using the open dynamic chamber technique. A flux chamber, manufactured according to specifications given in VDI 3880,^13^ was placed on top of the biofertilizer surface. A known flow of fresh air was flushed through the 0.5 m^2^ chamber (100 × 50 cm) using fans at a flow rate of 16 m^3^ h^–1^, and the methane concentration was measured continuously in the air flow from the chamber with a flame ionization detector together with a nonmethane hydrocarbon cutter which filters out other hydrocarbons than methane (EN ISO 25140:2010). The instrumentation included an FID BA3005 and a cutter Model 900 from JUM Engineering GmbH. The calibration gas had a concentration of 8000 ppm of CH_4_. The measured levels in combination with the air flow was combined to calculate CH_4_ fluxes. Normally, every third year, there is voluntary commitment by the plant to measure CH_4_ emissions from the storage tank; this is done using these types of chambers at 1–3 sites close to the tank wall. In this study, a more extensive measurement setup was used with a total measurement time of 5 h, allowing 10 chamber measurements at selected positions across the surface (Figure S2) in the range of 3–14 m from the concrete enclosure of the tank. Chamber placements were done using a crane mounted on a truck to reach chamber positions around the tank from its edge to about 1/3 of the distance to its center. Long tubes for inflow and outflow of air connected the chamber to a van at the edge of the tank containing the flame ionization detector. As for the seal at the base of the chamber, floats are regulated so that the hood sinks a bit below the surface (see Figure S9). Chamber sealing can be checked visually, although with greater difficulty for measurement points far away from the tank edge, which is one of the uncertainties with the chamber method. This was a much more thorough effort than previous flux chamber measurements at the plant, where normally only 2–3 chamber points were used, all close to the tank enclosure without using a crane. The greater effort in this study was for the purpose of reducing uncertainty from biased chamber placement and extrapolation from discrete measurements to the whole storage tank.

### Reference Measurements

Reference air samples were collected manually at selected positions around and inside the tank, as well as close to the camera, and at a distance far enough not to be affected by the tank emissions to capture the background CH_4_ level as an independent method for checking the range of CH_4_ concentrations. Each air sample was collected in three 60 mL plastic syringes (Becton, Dickinson) with Luer-Lock valves (180 mL in total). A 150 mL portion of this volume was used to flush a 22 mL glass vial precapped with a gas-impermeable butyl rubber septa (fluxing via two 0.4 mm diameter syringe needles, one from the syringes with samples and one for releasing outgoing sample). The last 30 mL sample was kept in the vial, and this gas was analyzed using a gas chromatograph (Agilent 6890 with a Poropak Q column and an FID (flame ionization detector)) to yield CH_4_ concentrations.

Figure S2 illustrates the location of our in situ air sampling, flux chamber positions, and remote sensing setup. In addition to this, we also used a FLIR GF320 IR camera, which is a conventional IR method often used to visualize CH_4_ leaks (but not to calculate fluxes as it cannot differentiate between different gases, such as CH_4_ and H_2_O). We also used laser-spectroscopy-based point measurements with high sensitivity (UGGA, Los Gatos Research) inside the front edge of the tank to verify CH_4_ concentrations at the surface and the top of the tank.

## Results

### Remote Sensing

#### Variable Mapping

**Figure 2:**
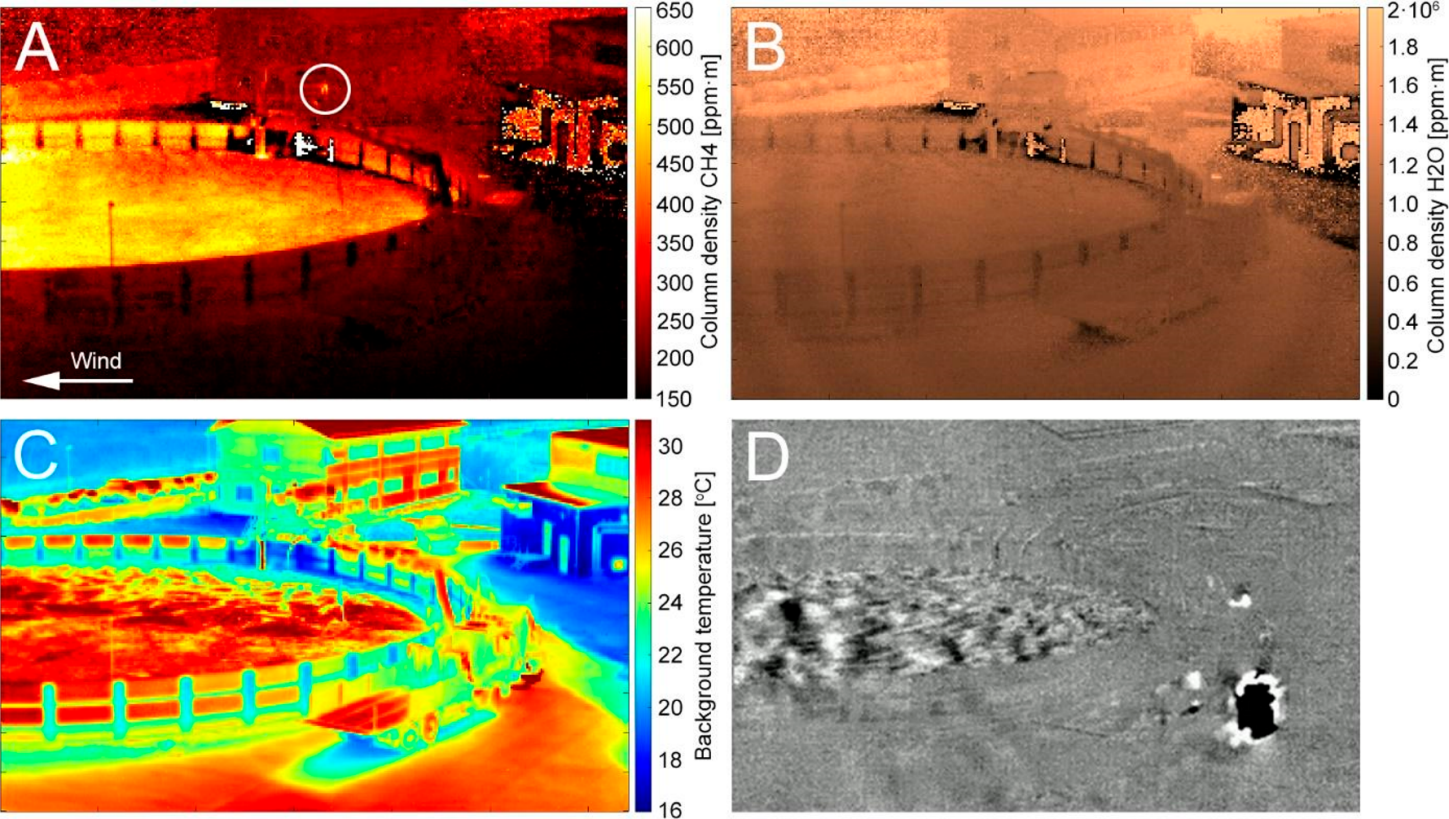
Example of calculated maps for the right half of the storage tank. (A) CH_4_ column density. The white circle marks a CH_4_ emission that was discovered close to the inlet to the storage tank. (B) H_2_O column density. (C) Background temperature. (D) A frame from a video sequence of differential IR images taken 1/4 s apart (showing changes in gas patterns in that time frame) used to calculate air motion. Note that the column density of H_2_O increases with background distance (B) while the highest CH_4_ column densities (A) are found toward the surface in the storage tank.

Figure 2 shows an example of calculated maps of CH_4_ and H_2_O column density, background temperature, and a frame from an air motion sequence calculated from the hyperspectral data. There is a clear CH_4_ gradient in the direction of the wind, and hotspots correlate with positions of higher surface temperature. Colder surfaces have lower CH_4_ emission, likely correlating with both lower heat conductivity and lower gas transmission related to thicker surface material or more sludge foam on the surface. Water vapor was homogeneously distributed inside and outside of the tank (panel B), indicating negligible H_2_O emissions from the tank. The temperature map (panel C) shows that there is a background–air temperature contrast of 5–15 °C toward the biofertilizer material, giving a high signal-to-noise ratio in all the maps. The biofertilizer material was thus much warmer than the surrounding air. The air temperature was also modeled from the spectra and showed values corresponding well with the air temperatures from the weather station.

#### Wind Speed

An example visualization of an air motion video created using the many time steps of an interferogram can be seen in SI Video 1. Cross-correlation of an area in a pair of frames then gives the positional shifts of features during a known time difference. SI Videos 1–3 show examples of air motion during different time periods, highlighting that the method can give clear videos for air motion visualizations and for calculations. Using the many time steps in a video, air velocities could automatically be calculated and plotted for the thousands of time steps that exist in an interferogram (example given in Figure S4), avoiding exposures in the interferogram with the strongest interference patterns. The average horizontal air motion inside the tank for the time period (19 data cubes, 14 min) used to calculate the CH_4_ flux (line L1 in Figure 1) was found to be 0.66 ± 0.03 m/s. This can be compared with the weather station having an average wind speed of 0.84 ± 0.04 m/s, also from the north but at one single point outside the tank. This corresponds with the expectations that the wind speeds are lower inside the tank and near surfaces, including wind shadowed parts in the scene, than outside the tank in free air where the weather station was located.

#### CH_4_ Flux from the Hyperspectral Approach

**Figure 3:**
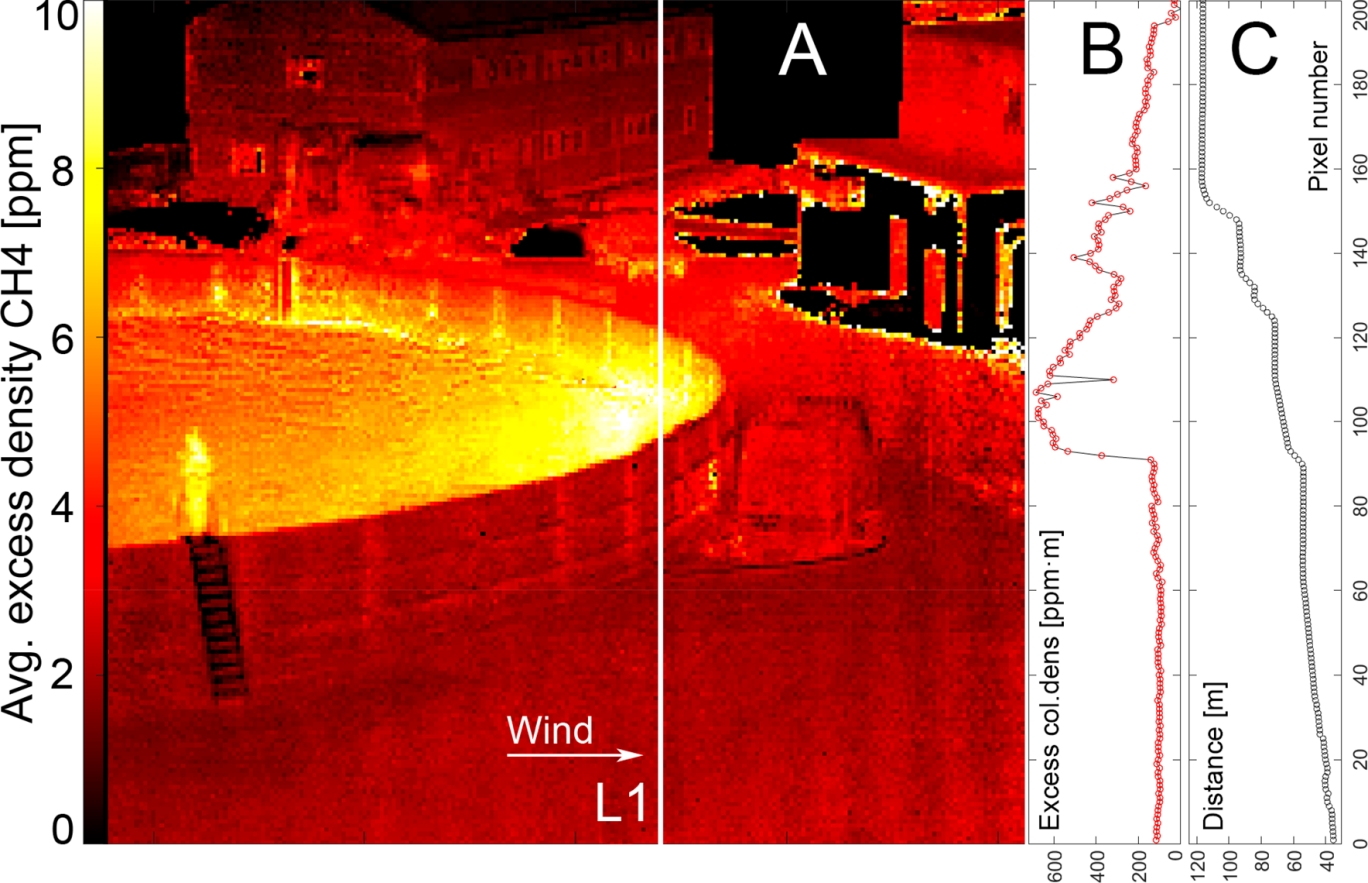
Results used for calculating the total CH_4_ tank flux during stable winds toward the right. (A) Excess CH_4_ map with cross section L1 marked, used for the mass balance calculations. (B) CH_4_ column density. Note that background levels are reached above pixel no. 195, showing that the full plume is accounted for despite plume propagation due to turbulence. (C) Background distance. Horizontal axes in panels B and C show positions along line L1, starting from the bottom of the field of view.

Figure 3 shows a CH_4_ excess concentration map calculated from excess column densities divided by background distance for each line of sight. Assuming a geometry where most of the CH_4_ is located inside and above the tank, and obtaining background CH_4_ concentrations from the lines of sight upwind and outside of the tank, a mass balance calculation through the vertical area outlined by line L1 yielded a total tank flux of 6.0 ± 1.3 kg CH_4_ h^–1^. The total flux was calculated by adding the flux for each pixel along the line, being long enough to include pixels from the front of the tank to pixels with lines of sight having backgrounds far behind and above the tank.

In order to estimate the uncertainties in this flux estimate and to verify that the tank edges and vertical motion of the gas did not affect the flux estimate significantly, we also estimated the flux from an area at the center of the tank (between lines L2 and L3, Figure S5) with the least influence from the edges from the camera point of view for a time period with stable winds toward the left (North). Corresponding average excess CH_4_ concentrations, column densities, and background distances (Figure S5) during stable winds (0.63 ± 0.03 m/s) gave an areal CH_4_ flux between lines L2 and L3 of 1.7 ± 0.4 mg CH_4_ m^–2^ s^–1^. The tank diameter was calculated from the geometry in the images using the LiDAR data to be 37.5 m, agreeing well with separate measurements using a laser range finder at the ground level. Given that the storage area has a surface of 1104 m^2^, the areal CH_4_ emission from the central tank area (Figure S5) extrapolated to the whole biofertilizer storage tank was 6.7 ± 1.6 kg CH_4_ h^–1^. This agrees with the total tank flux estimated directly from the L1 line cross section (Figure 3), which integrates the total flux in a more representative way by sampling the whole tank area.

### Traditional Methods

#### IR Camera

The FLIR GF320 IR leak detection camera along with the associated built-in method for visualizing emissions using differences between subsequent images was unable to detect any CH_4_ emissions from the biofertilizer tank (Figure S6). It was clear that such a narrow-band filter camera is primarily suitable for detecting point source emissions, such as leaks, having a high contrast in concentrations, high background–gas temperature contrast, and negligible water vapor emissions, as these can otherwise not be separated from the CH_4_ emissions.

#### Flux Chambers

In Table 1 the results from our chamber measurements are presented, with the total storage tank emission estimated using an average of all ten positions (each having a measurement time of 20 min). The biofertilizer surface was heterogeneous, as it had a large number of cracks, having what seemed to be a more wet and soft surface than the surrounding areas. There were also larger wet and soft surfaces with diameters less than 3 m close to the pumps at the inlet and at the loading location. At locations 1, 5, 6, and 8 (Figure S2B) the chamber was placed on cracked wet surfaces, while at the other locations the surfaces looked dry and intact. If we divide the locations into two categories, one group with dry and intact surfaces and one group with wet and cracked surfaces, we can calculate average fluxes of 12.2 ± 6.1 and 15 ± 6.8 kg of CH_4_ h^–1^, respectively. The total emission from the storage tank using the chamber method is estimated to be 13.3 ± 6.1 kg of CH_4_ h^–1^, corresponding to 1.4% of the total production capacity at the facility.

**Table 1:** Chamber Measurement Results^a^

position	time	CH_4_ (ppm)	total emission (kg CH_4_ h^–1^)	surface emission (g CH_4_ m^–2^ h^–1^)	volume emission (g CH_4_ m^–3^ h^–1^)
1	10:30	405	11	10.0	4.4
2	10:55	702	20	18.1	8.0
3	11:20	240	6.7	6.1	2.7
4	11:50	675	19	17.2	7.6
5	12:45	890	25	22.6	10.0
6	13:30	475	13	11.8	5.2
7	13:55	228	6.4	5.8	2.6
8	14:25	390	11	10.0	4.4
9	14:50	432	12	10.9	4.8
10	15:05	325	9.1	8.2	3.7
average			13.3 ± 6.1	12.1 ± 5.6	5.3 ± 2.5

a Start times, concentrations, and estimated emissions (total, surface specific, and digestate volume specific). The flow rate through the chamber was 16 m^3^ h^–1^.

### New Emission Discoveries

#### Flux Associated with Sludge Retrieval from the Tank

**Figure 4:**
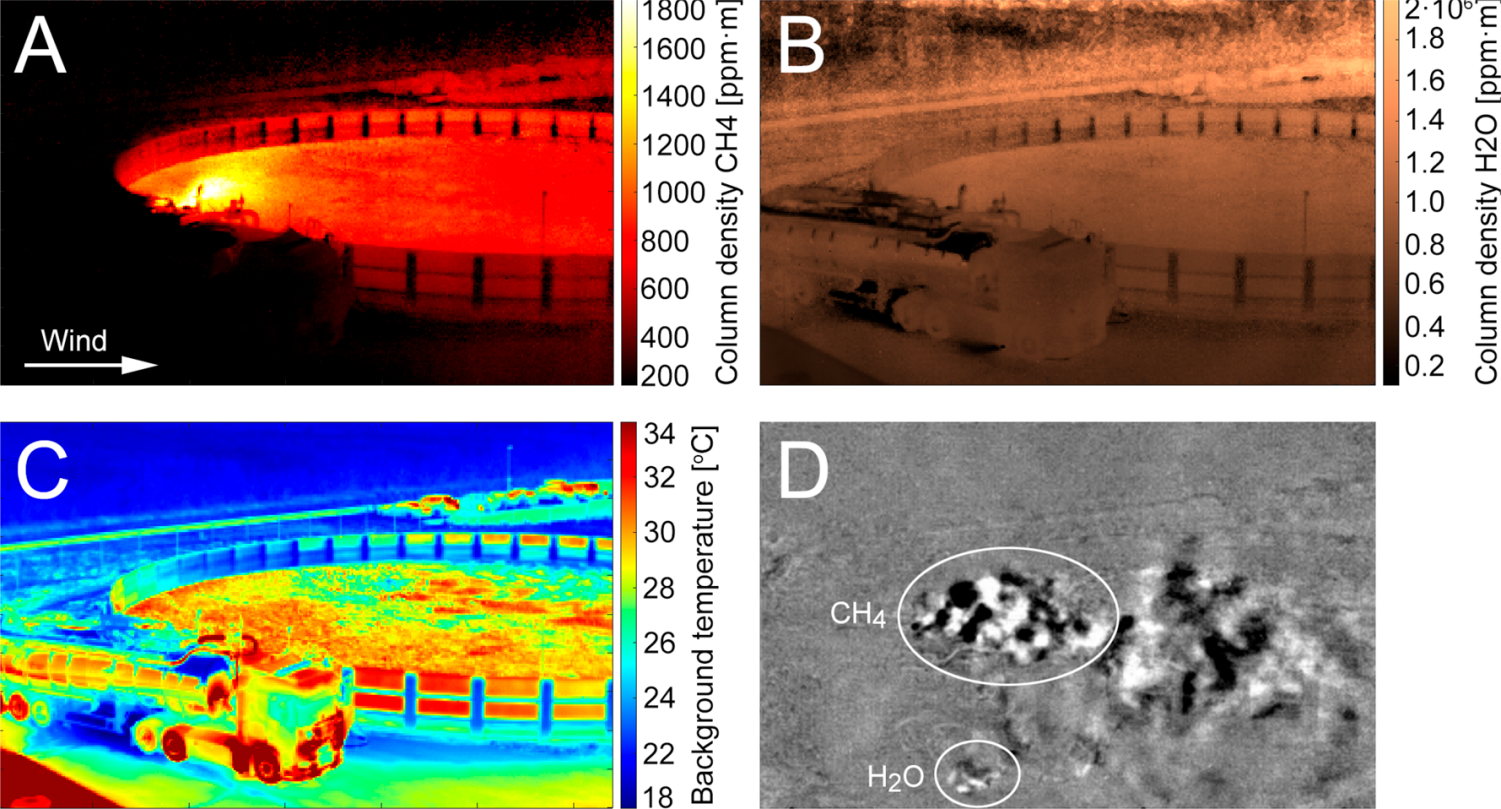
Maps during loading of the biofertilizer from the storage tank into a truck. The truck is located in the lower left part of the frame. (A) CH_4_ column density. (B) H_2_O column density. (C) Background temperature. (D) A frame from an emission video with high contrast made from subtracting infrared images adjacent in time, indicating large concentrations (very dark or light areas). From spectroscopic information, plumes of CH_4_ and H_2_O can be identified (see Figure S3A for an example spectrum). From the corresponding CH_4_ and H_2_O maps, it is clear that loading increases the CH_4_ emissions by a large amount (A), while H_2_O emissions are unaffected (B) except for a small addition from the exhaust pipe of the truck (D).

Trucks periodically retrieved biofertilizer from the left side of the storage tank (as seen from the camera point of view; Figures 1 and 4C), which caused increased levels of CH_4_ in the air above the tank. To avoid biased tank emissions, we generally avoided hyperspectral imaging during loading events. However, we did measure the CH_4_ flux from one of the loading events in order to compare the loading flux and the entire tank surface flux. The resulting maps are shown in Figure 4.

Warm water vapor emitted by the truck can be seen in these maps (Figures 4B and 4D), but it is easily separated from CH_4_ emissions in the spectroscopic modeling. In order to separate the loading and tank CH_4_ emissions, we used a concentration map created a few minutes prior to the loading event that could be subtracted from the map obtained during the event, giving an excess concentration map purely from the loading event. Flux calculations were made across the vertical line (Figure S7) perpendicular to the wind direction using six data cubes (3 min) that had stable wind conditions. The loading flux was calculated to be 3.1 kg CH_4_ h^–1^, showing that this flux cannot be neglected, and during loading times (lasting about 6 min), the flux from loading was roughly half of the tank flux (6.0 ± 1.3 kg CH_4_ h^–1^). A video showing the emissions from the truck and loading activity can be found in SI Video 2.

#### Flux Associated with Sludge Input to the Tank

Another emission source found from these measurements was prior to the point of inflow of biofertilizer material into the tank. This is connected to a sifting process step that removes plastic material in the biofertilizer right before being pumped into the tank. During sifting, the biofertilizer is mixed, causing gas to be emitted from the liquid. The mixed biofertilizer is also completely fresh and has not had time to cool (about 40 °C) compared to the temperature in the tank, making it more biologically active compared to the tank material. A hotspot can clearly be seen in the CH_4_ concentration map at the inlet of new material (Figure 2A) having a calculated flux of 16.8 g CH_4_ h^–1^ based on five data cubes (corresponding to a time period of 2.5 min with stable winds).

### Summary of Hyperspectral Assessments and Air Samples

We recalculated the hyperspectral column density maps into estimated average excess CH_4_ concentration maps (Figure S8) using background distances and geometry. A comparison with our manual air samples (Table S2) showed a good agreement in range, with the highest concentrations in the camera CH_4_ maps having concentrations of 65 ppm and the air samples with the highest concentrations (point b) being 62.9 and 77.9 ppm (depending on sampling height and time). This was supported by additional point measurements with a high-sensitivity ultraportable greenhouse gas analyzer (UGGA, Los Gatos Research) at the same sampling point, having large variations in the range 40–82 ppm with the highest concentrations close to the surface. The lowest concentrations inside the tank were found to be ∼25 and 27.2 ppm for the camera and air samples, respectively.

A summary of emission sources found with the hyperspectral camera is given in Table 2. It is clear that the tank surface is the dominating source, with the material inlet flux being 350 times smaller. However, at the times of the loading of material into trucks, the total facility flux can increase by about 50%.

**Table 2:** Summary of CH_4_ Emission Sources from the Hyperspectral Measurements

source	emission (kg CH_4_ h^–1^)
tank surface	6.0 ± 1.3
inlet	∼0.017
loading flux during event	∼3.1
loading flux daily average^a^	∼0.10

a Based on statistics for this facility, with eight loading events per day on average, each lasting 6 min.

## Discussion

The average production of CH_4_ gas at the biogas plant during 2 weeks before this study was 1330 N m^3^ h^–1^, corresponding to 950 kg CH_4_ h^–1^, which can be related to the tank surface emission estimated from the hyperspectral (6.0 ± 1.3 kg CH_4_ h^–1^) and chamber (13 ± 5.7 kg CH_4_ h^–1^) methods, implying emissions of 0.7% and 1.4% of the production, respectively. A previous study at the plant in September 2014^2^ showed an emission from the tank of 4.4 and 7.3 kg CH_4_ h^–1^ using static and dynamic chambers, respectively,^2^ but emissions can vary over time and measurements at different time points may not be directly comparable. Nevertheless, comparing the two studies (Table 3) illustrates similar orders of magnitude in fluxes. There was a 15% higher biogas production during our measurement day, possibly contributing to the higher chamber flux this day. Comparisons between the hyperspectral camera and chamber results indicates up to 100% higher tank flux estimates from the dynamic chamber method. We believe that this is in part due to chamber placement and the number of measurement positions used deciding how well the spatial variability was represented and possible chamber interactions with the sludge surface.

**Table 3:** Comparison of CH_4_ Emissions at the Linköping Plant between This Study and Reinelt et al.^2^

parameter	Reinelt et al.^2^	this study
storage volume (m^3^)	2000	2500
methane production (kg h^–1^)	823	950
Surface Emissions (kg CH_4_ h^–1^)		
static chamber	4.4	---
dynamic chamber	7.3	13.3
hyperspectral camera	---	6.0
Surface Flux (kg CH_4_ m^–2^ h^–1^)		
static chamber	4.1 (6.2^a^)	---
dynamic chamber	6.9	12.1
hyperspectral camera	---	5.4
Volume Flux (kg CH_4_ m^–3^ h^–1^)^b^		
static chamber	2.2 (3.3^a^)	---
dynamic chamber	3.7	5.3
hyperspectral camera	---	2.4

a Average of cracked surface layer.

b For the volume flux, we have used the sludge volume at the time of measurement.

The difference between the flux chamber results in 2014 and during our measurements illustrates the need to capture variability in time and not just short-term average values when trying to covert flux observations to general emissions factors. Clearly, CH_4_ emissions from open storage tanks depends on several influences like the digestate temperature (and hydraulic retention time in the AD process, filling level of the digestate vs content in the digestate, seasonal ambient conditions, etc.), which are changing during the year. This shows the importance of long-term studies or at least several measurement campaigns during a year to capture seasonal variability.^19,21^ We also note that fluxes from cracked surface areas in the Reinelt et al. study^2^ are clearly higher than other areas, which is in agreement with the tendency we observed in our study, showing the importance of chamber placements that are representative of the whole tank surface. There is also a possibility of changes in plant operation or substrate feeding between the two measurement campaigns (part of which can be seen by the change in CH_4_ production).

In a study of 23 biogas plants,^14^ CH_4_ losses varied in the range 0.4–14.9% of the biogas production, showing that there are large variations in facility emissions and that actions taken to reduce emissions can be effective. Such actions for methane emission reductions or recovery could include improving the digestion in biogas reactors, precooling of sludge before storage, or modifying open storage tanks to have a sealed, gastight cover. Performing before and after studies using methods similar to the ones in the current project will be needed to evaluate the effectiveness of emissions reductions.

The storage tank inlet has a negligible CH_4_ emission rate, but this still illustrates the capacity of the hyperspectral remote sensing method to also detect small unknown emission sources. It was unexpected to find that emissions from the biofertilizer loading into trucks were as large as 50% of the total storage flux during these loading events (6 min long). In order to estimate the importance of this loading flux, the frequency and duration of each event must be known, which will depend on the activity at each biogas plant. At this particular plant, on average there are eight loading events per day, giving a daily averaged loading flux of 0.10 kg CH_4_ h^–1^ (corresponding to 1.7% of the total storage tank flux and 0.31 kg CH_4_ per loading event). The importance of this previously unknown flux will depend on the loading frequency at a plant; in this case the loading emission during a year (2860 trucks × 6 min) is approximately 900 kg CH_4_ yr^–1^. Even if this flux is proportionally low compared to the total flux, there may be relatively simple ways to reduce such emissions by the design of a sludge transfer system from the tank to the truck.

The hyperspectral camera method has several advantages compared to traditional chamber methods including that it is noninvasive, allowing measurements during normal activity at a facility and not increasing the emissions through contact with the material in the tank, has the ability to cover the whole tank area, and is able to detect and quantify unknown emissions (such as the loading events and the mixing of material at the inlet). Disadvantages include the need for a high enough thermal contrast between the background and the emitted gas making the method weather dependent, potential difficulties finding a suitable place to set up the system (viewing angle relative to the sun and background used), the equipment being more expensive, extensive know-how needs to optimize calculations, and the fact that air motion can be difficult to map during periods with high turbulence and variable wind directions. The difficulty of mapping air motion close to the tank edges and part of the air inside the tank not being seen as it was hidden by its front edge can be reduced by measuring from a higher viewpoint or even from above using, e.g., a helicopter, or a drone with a high-enough payload capacity. In a future scenario, application of drone-mounted hyperspectral imaging systems may be realized when low-weight cameras are available. However, all gas leaving the tank, also from specific parts not directly visible with the camera, will pass the L1 line in Figure 1 and thereby be accounted for in the presented total tank flux assessment. With our approach, all detectable methane sources within the scene can be identified and, if not overlapping, quantified separately. Overlapping sources are not possible to distinguish. Therefore, our method allowed the detection of new sources such as emissions from the inlet and loading fluxes, along with the main emissions from the biofertilizer storage tank.

Comparing the variability in emissions for the hyperspectral method, the two areas used in the remote sensing gave rather similar total fluxes (6.0 ± 1.3 kg CH_4_ h^–1^ for line L1 and 6.7 ± 1.6 kg CH_4_ h^–1^ for the area between lines L2 and L3). Given the higher second estimate it is possible that measurements based on the most wind exposed parts (if higher wind speeds are assumed to increase emissions) could slightly overestimate total fluxes by not accounting for tank parts being less wind exposed, making whole tank integration desirable.

The camera measurements integrate over the total emitting surface and thereby primarily quantify variability over time in the total emission estimate, while the flux chamber measurements are more affected by variability both in space (between individual measurement locations) and in time (between individual measurement times over a longer total period). The greater variability among the flux chamber measurements (flux range 6.4–25 kg CH_4_ h^–1^) is therefore expected and makes the measurement strategy to obtain a total flux representing the whole tank important and challenging as a high workload and a long time period is needed for generating representative total flux estimates.

For large-scale emissions estimates from the biogas sector, results have to be comparable between plants, using techniques that give flux measurements representative of larger areas (emissions from digestate storage being one of the major GHG sources) and that allow detection and quantification of nonconventional and unexpected sources adding to the total flux (e.g., detecting unexpected leaks). Measurements with a potentially large under- or overestimation of plant fluxes will have a large impact on regional and global emission estimates from the biogas sector as the global biogas production is increasing rapidly with 17 400 large plants in the EU in 2015, 2100 in the United States in 2017, and 100 000 modern biogas plants in China in 2014.^15^ From this perspective, the hyperspectral camera approach demonstrated here appears to be a promising alternative for assessing emissions at high spatial detail, guiding mitigation efforts, and verifying mitigation success.

**Figure 5:**
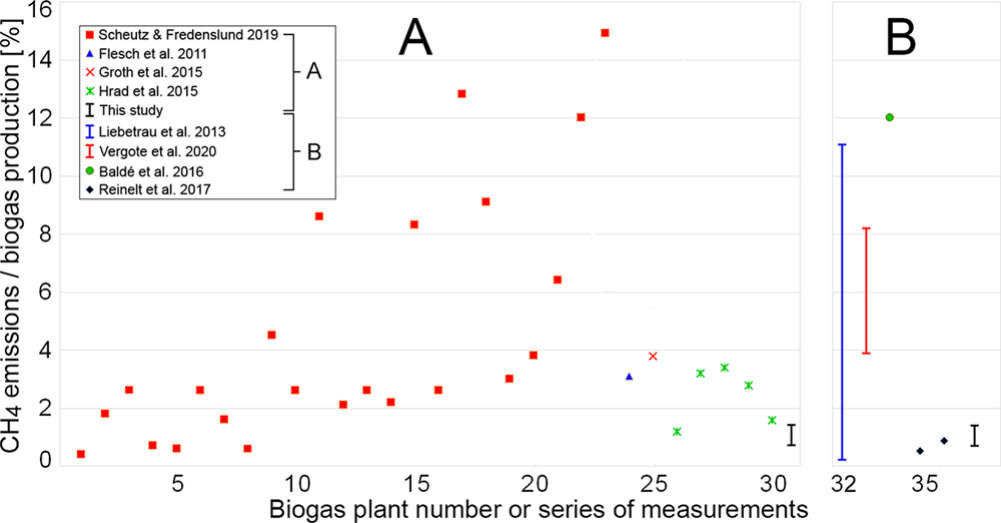
Comparison of CH_4_ emissions relative to gas production for biogas plants in the literature and our study. The different symbols indicate estimates for individual biogas plants while bars give ranges for a group of measurements or plants in a study. Measurements have been divided into full facility emission estimates (A) and emission estimates from digestate storage only (B), depending on what was reported. The emission estimates in our study are based on both the hyperspectral camera and chamber methods.

Comparing our measurements of CH_4_ digestate storage flux with other studies (Figure 5),^16,17^ we find that they are on the same level as other biogas plants on the low end of the previously reported fluxes, with digestate storage at some plants having much higher fluxes. It is presently unclear if the great variability in total and digestate storage among and within plants illustrated in Figure 5 depends on differences between method approaches or if it reflects true intrinsic variability. However, from our study we have noted that the total flux can vary considerably in time (e.g., trucks retrieving biofertilizer material), making the time of measurement and the identification of sources important for a correct emission estimate. For flux chambers, often only the digestate storage flux is included, while for methods measuring at a distance downwind from a plant (such as tracer dispersion), the flux source attribution is sometimes unclear.

Altogether the mini-review shown in Figure 5 highlights the importance of these emissions and measurements for a large number of plants. Although many of the measurements are of total emissions, several of the studies give digestate storage as one of the dominating sources.^2,14,18−21^ Scheutz and Fredenslund,^14^ for instance, find average total fluxes of 6.1% and 9.2% of the biogas production for plants without and with open digestate storage, respectively. They also found that the wastewater treatment biogas plants had higher emissions than agriculture plants, with average CH_4_ losses of 7.5% and 2.4%, respectively. Also, Liebetrau et al.^20^ finds digestate storage fluxes in the range of 0.22–11.2% in a study of 10 plants. If the data of Figure 5A are representative, approximately 4% of the biogas production is being emitted across these plants on average based on full facility CH_4_ emission estimates. Further, based on reported total European production of 18 billion m^3^ CH_4_/yr, representing 50% of the global production,^15^ emissions from storage tanks like those in focus here could correspond to more than 1 000 000 tonnes CH_4_/yr globally (1 Tg CH_4_/yr). Although representing a crude back-of-the-envelope estimate, 1 Tg CH_4_/yr is similar to 50% of the estimated global industrial emissions from fossil fuels or 25% of the global CH_4_ emissions in the transport sector.^22^ Hence it is critical to monitor and verify mitigation of these sources with effective and standardized or cross-validated methods. Importantly, accurate emission measurement methods provide tools to guide which mitigation efforts should be prioritized and allow the quantitative evaluation of different gas emission mitigation measures. This is key for the cost-effective reduction of GHG emissions. At the studied facility, the results of this paper have triggered intensive mitigation work and resulted in covering the studied tank and harvesting the CH_4_ formed upon sludge storage. Accordingly, the study including the hyperspectral assessment approach has already contributed to the reduction of some CH_4_ emissions.

## Supporting Information Available

Figures S1 (hyperspectral camera setup), S2 (manual air samples and flux chambers), S3 (hyperspectral example spectrum and residuals), S4 (wind speed plot), S5 (flux from an area at the center of the tank), S6 (imaging of biofertilizer storage tank using a narrow band IR camera), S7 (flux from loading of biofertilizer material), S8 (average excess CH_4_ surface concentrations), and S9 (flux chamber design); Tables S1 (terminology used in the main text) and S2 (manual air samples around and close to the biogas fertilizer storage area); and mass balance calculations (PDF)
Video 1 (air motion over tank, example 1) (MP4)
Video 2 (air motion when loading biofertilizer onto a truck) (MP4)
Video 3 (air motion over tank, example 3) (MP4)

## References

[ref1] Liebetrau J.; Clemens J.; Cuhls C.; Hafermann C.; Friehe J.; Weiland P.; Daniel-Gromke J. Methane emissions from biogas-producing facilities within the agricultural sector. Eng. Life Sci.10 2010, (6), 595–599. 10.1002/elsc.201000070.

[ref2] Reinelt T.; Delre A.; Westerkamp T.; Holmgren M.; Liebetrau J.; Scheutz C. Comparative use of different emission measurement approaches to determine methane emissions from a biogas plant. Waste Management 2017, 68, 173–185. 10.1016/j.wasman.2017.05.053. 28629708

[ref3] Rodhe L. K. K.; Ascue J.; Willén A.; Persson B. V.; Nordberg A. Greenhouse gas emissions from storage and field application of anaerobically digested and non-digested cattle slurry. Agriculture, Ecosystems & Environment. 2015, 199, 358–368. 10.1016/j.agee.2014.10.004.

[ref4] Willén A.; Rodhe L.; Pell M.; Jönsson H. Nitrous oxide and methane emissions during storage of dewatered digested sewage sludge. Journal of Environmental Management 2016, 184, 560–568. 10.1016/j.jenvman.2016.10.025. 27784582

[ref5] Michael Fredenslund A.; Gudmundsson E.; Maria Falk J.; Scheutz C. The Danish national effort to minimise methane emissions from biogas plants. Waste Management 2023, 157, 321–329. 10.1016/j.wasman.2022.12.035. 36592586

[ref6] Bakkaloglu S.; Cooper J.; Hawkes A. Methane emissions along biomethane and biogas supply chains are underestimated. One Earth 2022, 5, 724–736. 10.1016/j.oneear.2022.05.012.

[ref7] Mønster J.; Samuelsson J.; Kjeldsen P.; Rella C.; Scheutz C. Quantifying robomethane emission from fugitive sources by combining tracer release and downwind measurements – a sensitivity analysis based on multiple field surveys. Waste Management 2014, 34, 1416–1428. 10.1016/j.wasman.2014.03.025. 24759753

[ref8] Mønster J.; Kjeldsen P.; Scheutz C. Methodologies for measuring fugitive methane emissions from landfills - A review. Waste Management 2019, 87, 835–859. 10.1016/j.wasman.2018.12.047. 30660403

[ref9] Burgués J.; Marco S. Environmental chemical sensing using small drones: A review. Sci. Total Environ. 2020, 748, 141172 10.1016/j.scitotenv.2020.141172. 32805561

[ref10] Gålfalk M.; Olofsson G.; Crill P.; Bastviken D. Making methane visible. Nat. Clim. Chang. 2016, 6, 426–430. 10.1038/nclimate2877.

[ref11] Gålfalk M.; Olofsson G.; Bastviken D. Approaches for hyperspectral remote flux quantification and visualization of GHGs in the environment. Remote Sensing of Environment 2017, 191, 81–94. 10.1016/j.rse.2017.01.012.

[ref12] Gålfalk M.; Bastviken D. Remote sensing of methane and nitrous oxide fluxes from waste incineration. Waste Management 2018, 75, 319–326. 10.1016/j.wasman.2018.01.031. 29397278

[ref13] Engl. VDI/DIN-Kommission Reinhaltung der Luft (KRdL) - Normenausschuss. Olfactometry - Static sampling; VDI 3880. https://www.vdi.de/en/home/vdi-standards/details/vdi-3880-olfactometry-static-sampling (accessed 2023-12-18).

[ref14] Scheutz C.; Fredenslund A. M. Total methane emission rates and losses from 23 biogas plants. Waste Management 2019, 97, 38–46. 10.1016/j.wasman.2019.07.029. 31447025

[ref15] Scarlat N.; Dallemand J.-F.; Fahl F. Biogas: Developments and perspectives in Europe. Renewable Energy 2018, 129, 457–472. 10.1016/j.renene.2018.03.006.

[ref16] Flesch T. K.; Desjardins R. L.; Worth D. Fugitive methane emissions from an agricultural biodigester. Biomass Bioenerg. 2011, 35, 3927–3935. 10.1016/j.biombioe.2011.06.009.

[ref17] Groth A.; Maurer C.; Reiser M.; Kranert M. Determination of methane emission rates on a biogas plant using data from laser absorption spectrometry. Bioresour. Technol. 2015, 178, 359–361. 10.1016/j.biortech.2014.09.112. 25446786

[ref18] Hrad M.; Piringer M.; Huber-Humer M. Determining methane emissions from biogas plants – Operational and meteorological aspects. Bioresour. Technol. 2015, 191, 234–243. 10.1016/j.biortech.2015.05.016. 26000833

[ref19] Baldé H.; VanderZaag A. C.; Burtt S. D.; Wagner-Riddle C.; Crolla A.; Desjardins R. L.; MacDonald D. J. Methane emissions from digestate at an agricultural biogas plant. Bioresour. Technol. 2016, 216, 914–922. 10.1016/j.biortech.2016.06.031. 27323243

[ref20] Liebetrau J.; Reinelt T.; Clemens J.; Hafermann C.; Friehe J.; Weiland P. Analysis of greenhouse gas emissions from 10 biogas plants within the agricultural sector. Water Sci. Technol. 2013, 67, 1370–1379. 10.2166/wst.2013.005. 23508164

[ref21] Vergote T. L.I.; Bode S.; De Dobbelaere A. E.J.; Buysse J.; Meers E.; Volcke E. I.P. Monitoring methane and nitrous oxide emissions from digestate storage following manure mono-digestion. Biosystems Engineering 2020, 196, 159–171. 10.1016/j.biosystemseng.2020.05.011.

[ref22] Saunois M.; Stavert A. R.; Poulter B.; et al. The Global Methane Budget 2000–2017. Earth Syst. Sci. Data 2020, 12, 1561–1623. 10.5194/essd-12-1561-2020.

[ref23] Galfalk M.; Paledal S. N.; Sehlen R.; Bastviken D. Ground-based remote sensing of CH_4_ and N_2_O fluxes from a wastewater treatment plant and nearby biogas production with discoveries of unexpected sources. Environmental Research 2022, 204, 111978 10.1016/j.envres.2021.111978. 34480946

