# Supplementary material for: Measurements of Methane Emissions from a Biofertilizer Storage Tank Using Ground-Based Hyperspectral Imaging and Flux Chambers
Source: Environ Sci Technol. 2024 Feb 14;58(8):3766–75. doi: 10.1021/acs.est.3c06810 (PMC10902839; doi:10.1021/acs.est.3c06810)
Supplement: Supplementary file 1 — es3c06810_si_001.pdf [file es3c06810_si_001.pdf]

Supporting Information for "Measurements of methane emissions from a biofertilizer storage tank using ground-based hyperspectral imaging and flux chambers"

Magnus Gålfalk[*,1], Sören Nilsson Påledal[2], Johan Yngvesson[3], David Bastviken[1]
* corresponding author: magnus.galfalk@liu.se
[1] Department of Thematic Studies – Environmental Change, Linköping University, Sweden.
[2] Tekniska Verken AB, Linköping, Sweden.
[3] RISE, Research Institutes of Sweden.

Contents of this file
Pages: 11
Figures: 8 (S1-S9)
Tables: 2 (S1-S2)

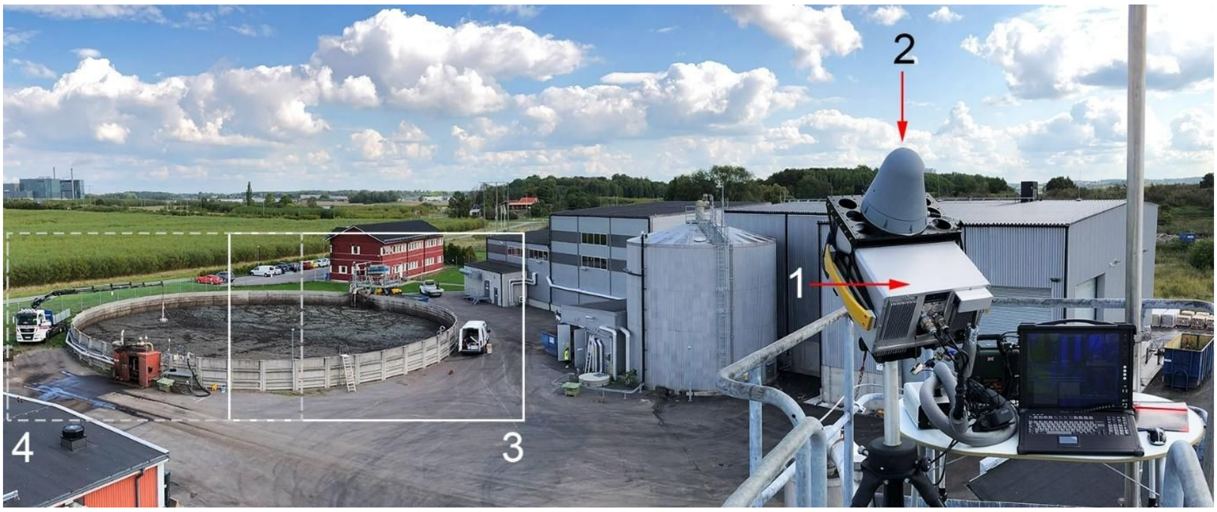

Figure S1. The hyperspectral camera setup at the biofertilizer storage tank. The measurements were made from an altitude of 12.7 m above the tank with background distances in the range 46 – 101 m. Mounted on top of the instrument (1) is a Lidar (2) that simultaneously maps distances in the same field of view (3, 4) as the spectroscopic images.

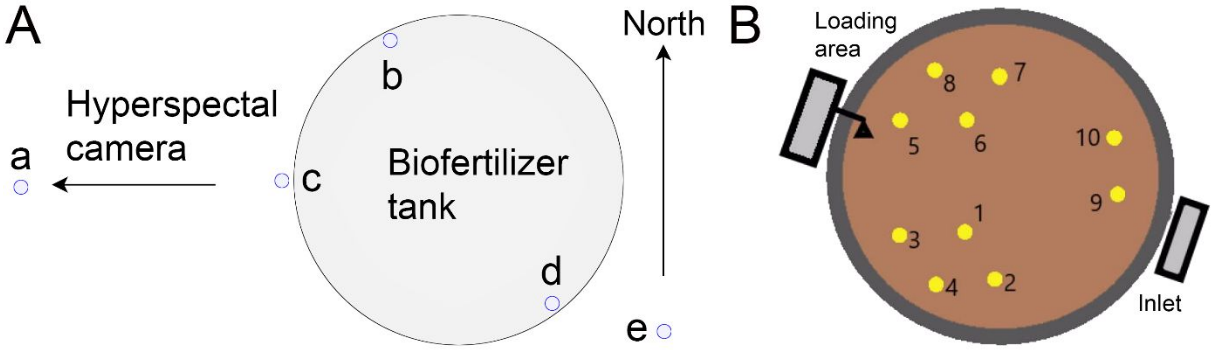

Figure S2. Illustration of geometry and measurements. (**A**) The hyperspectral camera's position relative to the biofertilizer storage tank and the sites of the manual air samples given with letters a-e. Sample sites a (camera) and e (background CH_4 level) are much further from the tank than they appear in this compact illustration. (**B**) Chamber positions for the traditional flux chamber measurements. The inlet of biofertilizer into the tank and the loading area for pumping of material into trucks are marked.

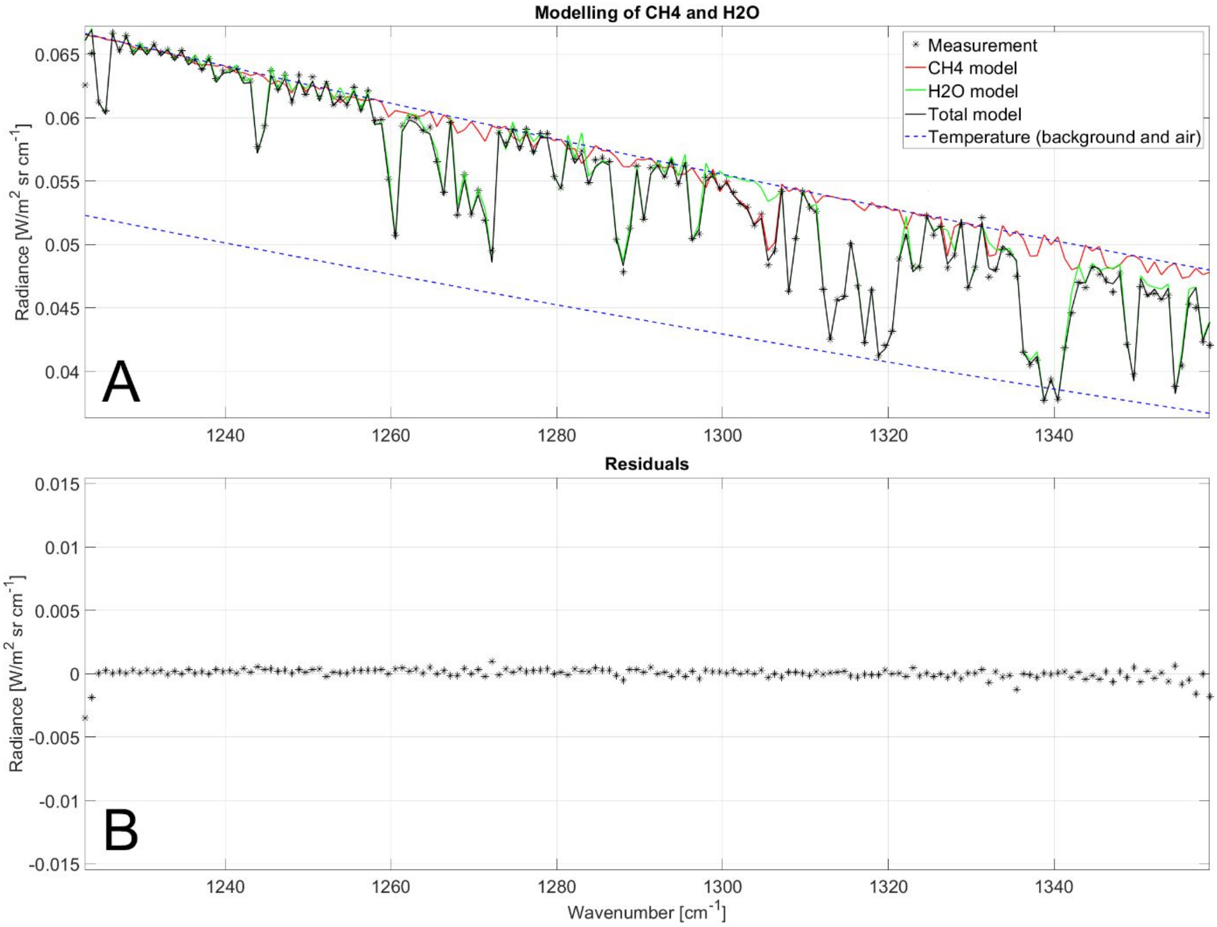

Figure S3. Example spectrum (**A**) and residuals (**B**) for a line of sight towards the center of the storage tank using the hyperspectral camera, with best-fit models plotted for CH_4, H_2O and total absorption. Background and air temperatures are 30.5 and 18.5 ^oC, respectively.

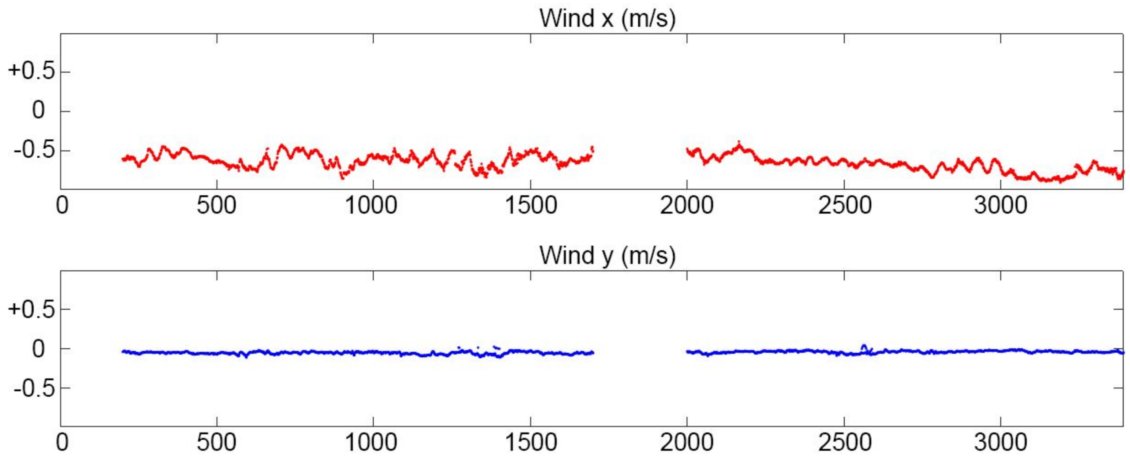

Figure S4. Example of a wind speed plot calculated from one of the data cubes (3 500 measurements in 30 seconds) having a horizontal wind speed of 0.63 ± 0.14 m/s (upper panel) while the vertical air motion was found to be negligible (lower panel).

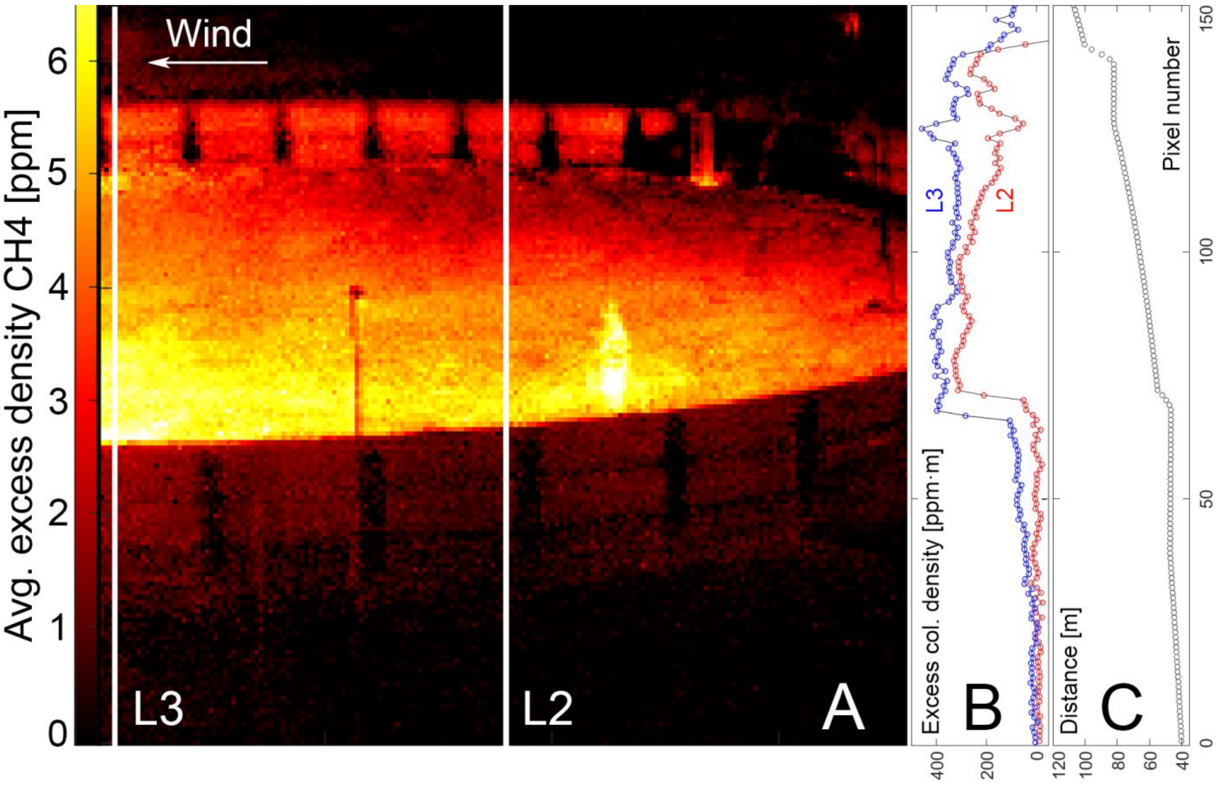

Figure S5. Visualization of excess CH_4 (mixing rations above the ambient background) with cross sections L2 and L3 marked, used for mass balance calculations in the L2 (inflow) to L3 (outflow) region during steady southern winds. Results used for estimating the total CH_4 tank flux during stable winds towards the left using a central area of the tank surface for error estimation, and to test importance of proximity to the tank edges for flux calculations. (**A**) Excess CH_4 map with cross sections L2 and L3 marked, used for mass balance calculations during steady southern winds. (**B**) CH_4 column density. (**C**) Background distance. Horizontal axes in panels B-C show positions along lines L2 and L3, starting from the bottom of the field of view.

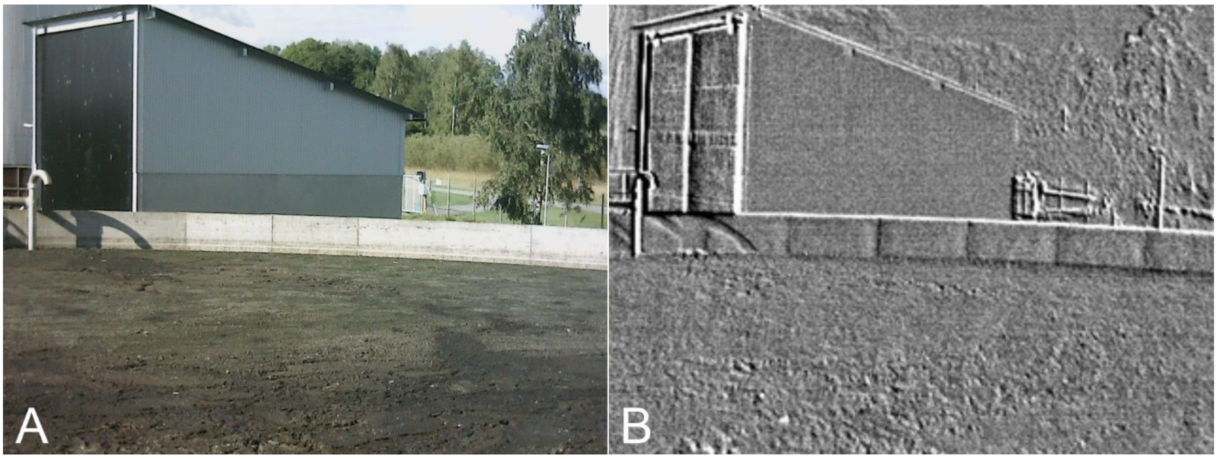

Figure S6. Imaging of the tank using a narrow band IR camera (FLIR GF320) as an illustration of what can be seen with the most common method for leak detection. A visual image (**A**) compared with a differential IR image (**B**) only shows physical structures from moving of the camera. Traditional IR imaging thus have difficulties visualizing CH_4, as extended homogeneous emissions will only result in a weak gradient in concentrations, this is however not a problem using hyperspectral imaging with spectroscopic modeling (Fig. S3).

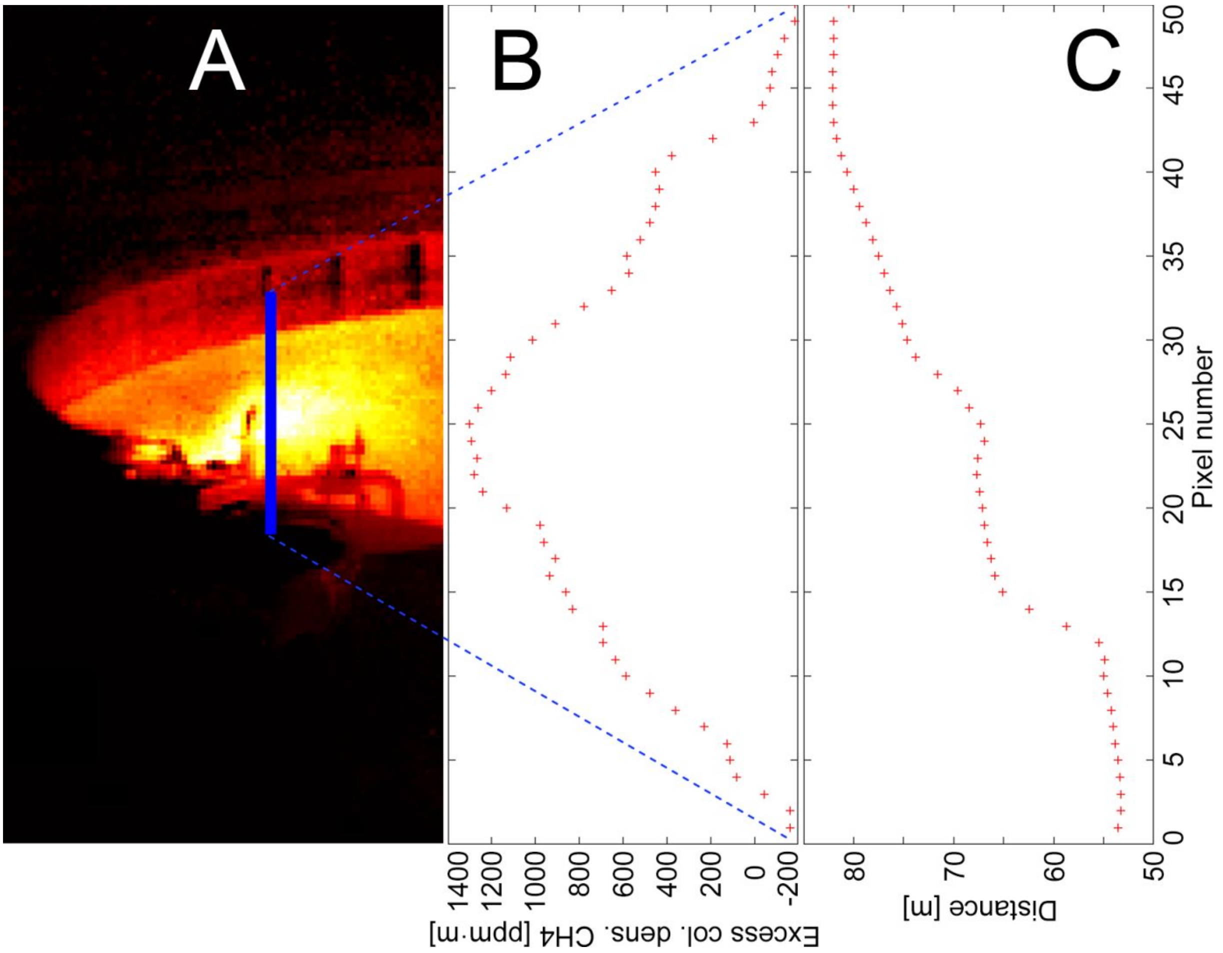

Figure S7. (**A**) Cross section used for calculations of the emission plume from biofertilizer loading marked with a blue line. **(B)** CH_4 excess column density. (**C**) Background distance. The horizontal axes in panels B-C show positions along the blue line in panel A, starting from the bottom.

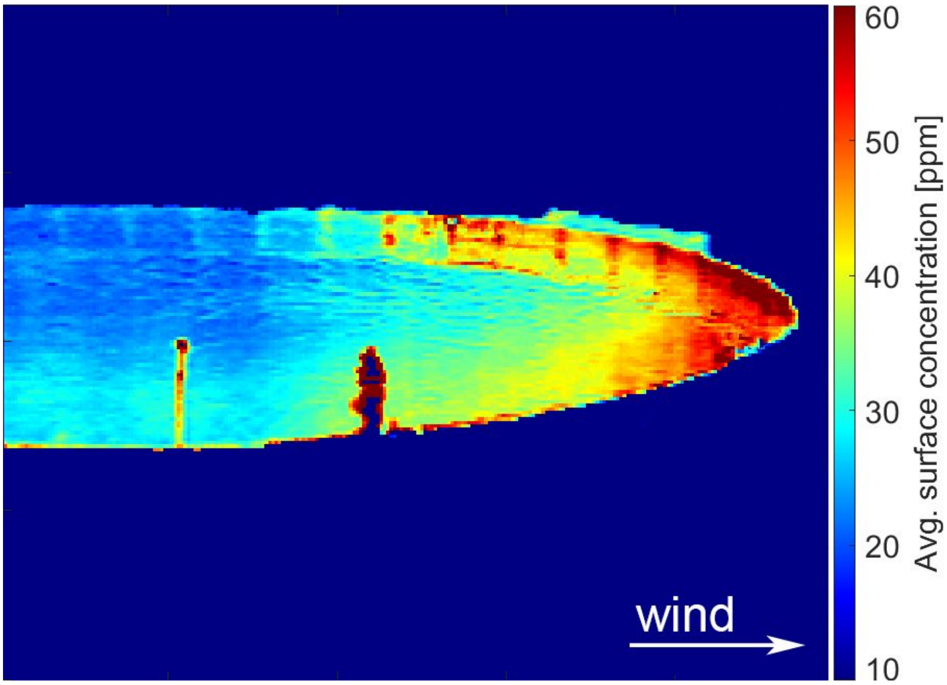

Figure S8. Map of average excess CH_4 surface concentrations (above background level) inside the open storage tank calculated by assuming that all excess CH_4 along each line of sight is located inside the tank. There is a clear concentration gradient in the direction of the wind, as might be expected, with concentrations in the range 25 – 65 ppm.

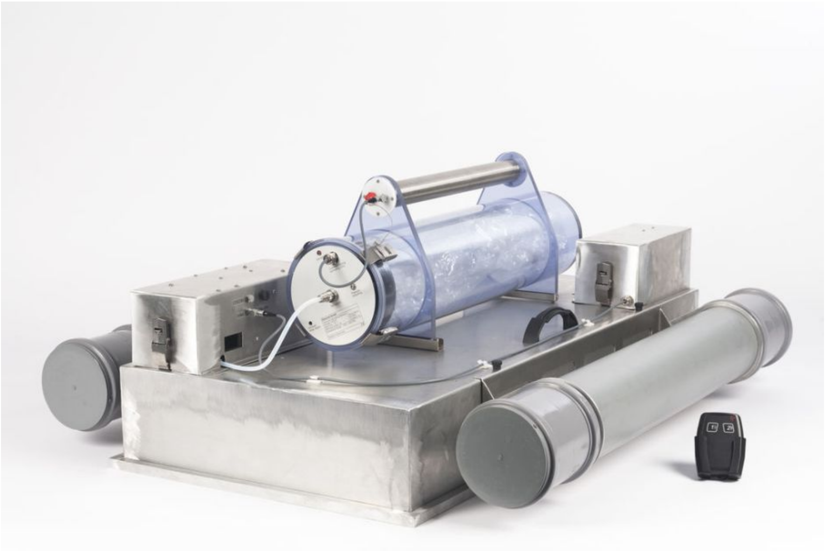

Figure S9. Flux chamber design used in this study. Sealing is achieved by adjusting the two floats so that the chamber floated with its edges below the sludge surface. This was confirmed by visual inspection.

Terminology

Table S1. Terminology used in the main text

Terminology	Explaination
Data cube	A file containing a series of measurements from a hyperspectral camera, that can be used to calculate one

	spectrum for each pixel in an image. In our setup a maximum resolution of 320 x 256 pixels.
Column density *N*	The amount of a gas from the instrument to the background, either expressed as mass per surface area perpendicular to the line of sight (e.g. g·cm^{-2}) or in ppm·m
Column density map *N(x,y)*	A map of total gas amount (from the camera to the background) showing the gas distribution
Background distance *z*	Distance from the camera lens to a solid background, different for each line of sight.
Flux	Emission from a source (kg·h^{-1})
Hyperspectral camera	An imaging Fourier transform spectrometer that takes thousands of exposures in one measurement – that can later be used to calculate spectroscopic maps
Lidar	A laser-based instrument for making background distance maps using the time that light takes to reflect back to a detector
Flux chamber	A chamber that is placed on top of a surface, measuring the emitted gas from that surface area

Table S2. Manual air samples around and close to the biogas fertilizer storage area. Sampling points are shown in Fig. S2A.

Measurement	Conc. CH_4 (ppm)
a	4.83
b (sample 1)	62.90
b (sample 2)	77.92
c	31.72
d	27.18
e	9.06

Mass balance calculation

The method for making CH_4 column density maps using spectroscopic modeling with the hyperspectral camera, and simultaneous air motion visualization, is given in detail in Gålfalk et al. (2017). Methane fluxes are then calculated from vertical lines in CH_4 column density

maps and average wind speed during the measurement period using mass balance. For the whole biofertilizer tank this means using line L1 in Fig. 1, which represents a vertical surface which all emitted gas from the tank passes through, relating this to the incoming air to the left of the tank (having ambient CH_4 levels). Average wind speed v_{avg} is calculated from air motion at the center of the tank (using videos of background subtracted IR sequences, showing plumes of CH_4 and H_2O moving across the tank; see example videos in the SI).

For each line of sight in the CH_4 column density map N(x,y) the volume measured is in the form of a pyramid with its top point located on the camera, the base located at the background, a height equal to the background distance *z*, and an angle equal to the field of view of a camera pixel (1.36 mrad x 1.36 mrad). By calculating the distance to the front of the tank (*z1*) and remaining distance to the background inside/across the tank (*z2*) the average excess CH_4 concentration in the air from the tank at coordinate (x,y) can be calculated as:

$$\rho_{excess}(x,y) = N(x,y) / z2$$

where N(x,y) and $\rho_{excess}(x,y)$ are in units ppm·m and ppm, respectively.

Converting excess CH_4 density from ppm to g/m3 we now have:

$$\rho_{excess}(x,y)\ [g/m3] = (\rho_{excess}(x,y)\ [ppm] \cdot P \cdot 10^{-6} / (R \cdot T)) \cdot M_{CH4}$$

where P is the standard air pressure (101.323 Pa), R the universal gas constant (8.314 J K^{-1} mol^{-1}), T the gas temperature in Kelvin, and M_{CH4} the molar mass of methane (16.0313 g/mol).

By calculating the volume of air for a pixel from the front of the tank to the background $V_2 = A_2 \cdot v_{avg}$, where A2 is the cross section perpendicular to the wind, we can now integrate the total flux from the tank by adding all pixels along the line L1 (see Fig. 3) using the different excess CH_4 concentrations and background distances of each pixel at coordinate y:

$$F_{tot} = \Sigma(\rho_{excess}(y) \cdot A_2(y) \cdot v_{avg})$$

where F_{tot} is in the units g CH_4 s^{-1}

References (SI)

Gålfalk, M., Olofsson, G., & Bastviken, D. (2017). Approaches for hyperspectral remote flux quantification and visualization of GHGs in the environment. Remote Sensing of Environment, 191, 81-94. doi:https://doi.org/10.1016/j.rse.2017.01.012
